# Inerolysin and vaginolysin, the cytolysins implicated in vaginal dysbiosis, differently impair molecular integrity of phospholipid membranes

**DOI:** 10.1038/s41598-019-47043-5

**Published:** 2019-07-23

**Authors:** Tadas Ragaliauskas, Milda Plečkaitytė, Marija Jankunec, Linas Labanauskas, Lina Baranauskiene, Gintaras Valincius

**Affiliations:** 1 Institute of Biochemistry, Life Sciences Centre, Vilnius University, Saulėtekio al. 7, LT-10257 Vilnius, Lithuania; 2 Institute of Biotechnology, Life Sciences Centre, Vilnius University, Saulėtekio al. 7, LT-10257 Vilnius, Lithuania; 3 Center for Physical Sciences and Technology, Saulėtekio al. 3, Vilnius, LT-10257 Lithuania

**Keywords:** Surface spectroscopy, Membrane structure and assembly, Atomic force microscopy, Infectious-disease diagnostics

## Abstract

The pore-forming toxins, inerolysin (INY) and vaginolysin (VLY), produced by vaginal bacteria *Lactobacillus iners* and *Gardnerella vaginalis* were studied using the artificial cholesterol-rich tethered bilayer membranes (tBLMs) by electrochemical techniques. The electrochemical impedance spectroscopy (EIS) of tBLMs attested for the toxin-induced impairment of the integrity of phospholipid membranes. This observation was in line with the atomic force microscopy data demonstrating formation of oligomeric protein assemblies in tBLMs. These assemblies exhibited different morphologies: VLY mostly formed complete rings, whereas INY produced arciform structures. We found that both EIS (membrane damage) and the surface plasmon resonance (protein binding) data obtained on tBLMs are in-line with the data obtained in human cell lysis experiments. EIS, however, is capable of capturing effects inaccessible for biological activity assays. Specifically, we found that the INY-induced damage of tBLMs is nearly a linear function of membrane cholesterol content, whereas VLY triggered significant damage only at high (50 mol%) cholesterol concentrations. The observed differences of INY and VLY activities on phospholipid membranes might have clinical importance: both toxin-producing bacteria have been found in healthy vagina and dysbiosis, suggesting the need for adaptation at different vaginal conditions. Our results broaden the possibilities of application of tBLMs in medical diagnostics.

## Introduction

Cholesterol-dependent cytolysins (CDCs) belong to a family of pore-forming toxins produced by many Gram-positive pathogenic bacteria and some gram-negative commensals^1,2^. The majority of these toxins are secreted and their pore-forming activity is fully dependent on host cell membrane cholesterol^3–5^. CDCs bind to the cell membrane, form large oligomeric complexes and subsequently inserted into the bilayer to form aqueous pores^6–8^. Despite the structural similarity of CDCs^9–11^, a distinct group emerged which binds to human glycosyl-phosphatidylinositol (GPI)-anchored membrane complement glycoprotein CD59^12–14^. Cytolysins produced by pathogenic bacteria participate in various stages of disease promoting bacterial invasion and infection^15^.

The activity of CDCs including some aspects of pore-forming mechanism have been studied using human and non-human cells, cell lines and polarized human vaginal tissue model^16–18^. The alternative synthetic biomimetic models have been proposed to simplify and accelerate the measurement of activity and elucidate the processes that occur on the membrane surfaces. Tethered lipid bilayer membranes (tBLMs) represent an artificial phospholipid membrane, which has been used to study protein-membrane interactions^19,20^. Availability to incorporate cholesterol into tBLMs makes them even more attractive for bioanalytical studies due to their similarity to the natural biological membranes^21^. The defects or water-filled pores in tBLMs induced by pore-forming toxins can be detected in real-time by electrochemical impedance spectroscopy (EIS)^22,23^ and visualized by atomic force microscopy (AFM)^24^.

In this study, we investigate binding and reconstitution of cholesterol-dependent cytolysins, inerolysin (INY) and vaginolysin (VLY), into the cholesterol-rich tBLMs at various pH as well as cholesterol amount embedded into the lipid bilayer. Inhabitant of human vaginal microbiota bacteria *Lactobacillus iners*, produces INY, which sole receptor, as most of CDCs, is cholesterol^16^. Another vaginal bacteria *Gardnerella vaginalis* secretes CDC toxin vaginolysin (VLY), which like intermedilysin (ILY) and lectinolysin use CD59 as their receptor^13^. Unlike ILY, VLY is able to lyse non-human cells, but the efficiency is approximately 100-fold less than that of human cells anchoring CD59 on their surface^17^. The frequent inhabitants of the human vagina, bacteria *L. iners* and *G. vaginalis*, were detected in normal vaginal microbiota as well as during dysbiosis^25–27^. Thus, both cytolysins are affected by various vaginal conditions characterized also by different pH value^28^. Previously reported pH modulation of both INY and VLY activity detected on mammalian erythrocytes^16^ was confirmed in this study by tBLMs, suggesting that artificial membranes tethered to a surface is a suitable model for detection of various groups of CDCs with perspective application in real clinical samples.

## Results

### Hemolytic activity of cytolysins at different pH

It was reported that INY and VLY showed their maximum activity on human erythrocytes at different pH^16^. Consistent with prior findings^16^ the hemolytic activity of INY dramatically decreased at pH ≥ 7.0, whereas activity of VLY declined at pH 4.5 (Table 1). To exclude the possibility that the observed pH-dependent activity of toxins is not related to protein unfolding, we used a fluorescent probe ANS (Supplementary File S1). In parallel, the thermal unfolding transition curves were measured on a Jasco J-815 circular dichroism spectropolarimeter (Supplementary Fig. S1). According to the test both INY and VLY remained stable within pH range from 4.5 to 7.0 and temperature range from 30 to 40 °C.

**Table 1:** Hemolytic activity of INY and VLY at different pH.

pH	HD_50_, pM	
	INY	VLY
4.5	18 ± 1.2	>200
5.0	17 ± 1	5.6 ± 0.5
5.5	28 ± 1.5	5.7 ± 0.6
6.0	24 ± 1	5.3 ± 0.8
6.5	280 ± 5	5.9 ± 0.7
7.0	>880	6.3 ± 0.5
7.5	NA	6.2 ± 0.5

### Effect of pH on binding of cytolysins to tBLMs

**Figure 1:**
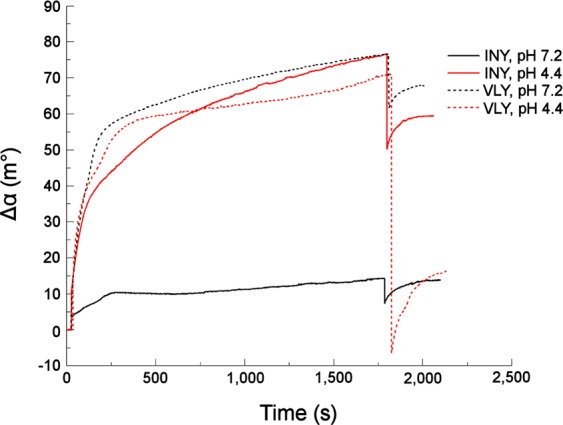
SPR angle traces produced by binding of INY (solid line) and VLY (dotted line) to cholesterol-rich tBLMs at pH 4.4 (red line) and 7.2 (black line). VLY and INY concentrations in solution were 20 nM and 5 nM, respectively. tBLMs were produced using DOPC/cholesterol multilamellar vesicles (MLVs) with a molar ratio of 50/50.

We next examined binding of INY and VLY to cholesterol-rich tBLMs containing DOPC/cholesterol at a molar ratio of 50/50 at pH 4.4 and 7.2 (Fig. 1). The surface plasmon resonance (SPR) signal increased following addition of toxins to tBLMs and reached the SPR angle shift Δα ≈ 65–70 millidegrees (m°) at pH 4.4 (Fig. 1) for both proteins.

It is worth mentioning that the concentrations of proteins triggering similar shifts were significantly different: 5 nM INY and 20 nM VLY. Also, the SPR shits exhibited quite different responses to replacement of protein-containing solution with pure buffer. Specifically, in case of VLY, rinsing the SPR cell with protein-free buffer (pH 4.4) triggered a remarkable decrease of the SPR shift. A significantly smaller decrease was detected upon removal of the INY from the cell at pH 4.4 (Fig. 1). At pH 7.2, the shift of the SPR angle induced by INY was much smaller (Δα ~ 15 m°) than the shift induced by VLY (Δα ~ 70 m°) (Fig. 1). In both cases, rinsing the cell with the protein-free buffer resulted in relatively small changes of the SPR profile.

### Inerolysin-induced dielectric damage of tBLMs is pH-dependent

The pore-forming toxins induce changes in the electrochemical impedance spectra (EIS) of tBLMs containing cholesterol^29–31^. It was demonstrated that those changes were consistent with the formation of water-filled pores in the membranes^23^. A prior report demonstrated that VLY induces dielectric damage in tBLMs even in the absence of CD59, while the presence of cholesterol remains unconditional^30^.

**Figure 2:**
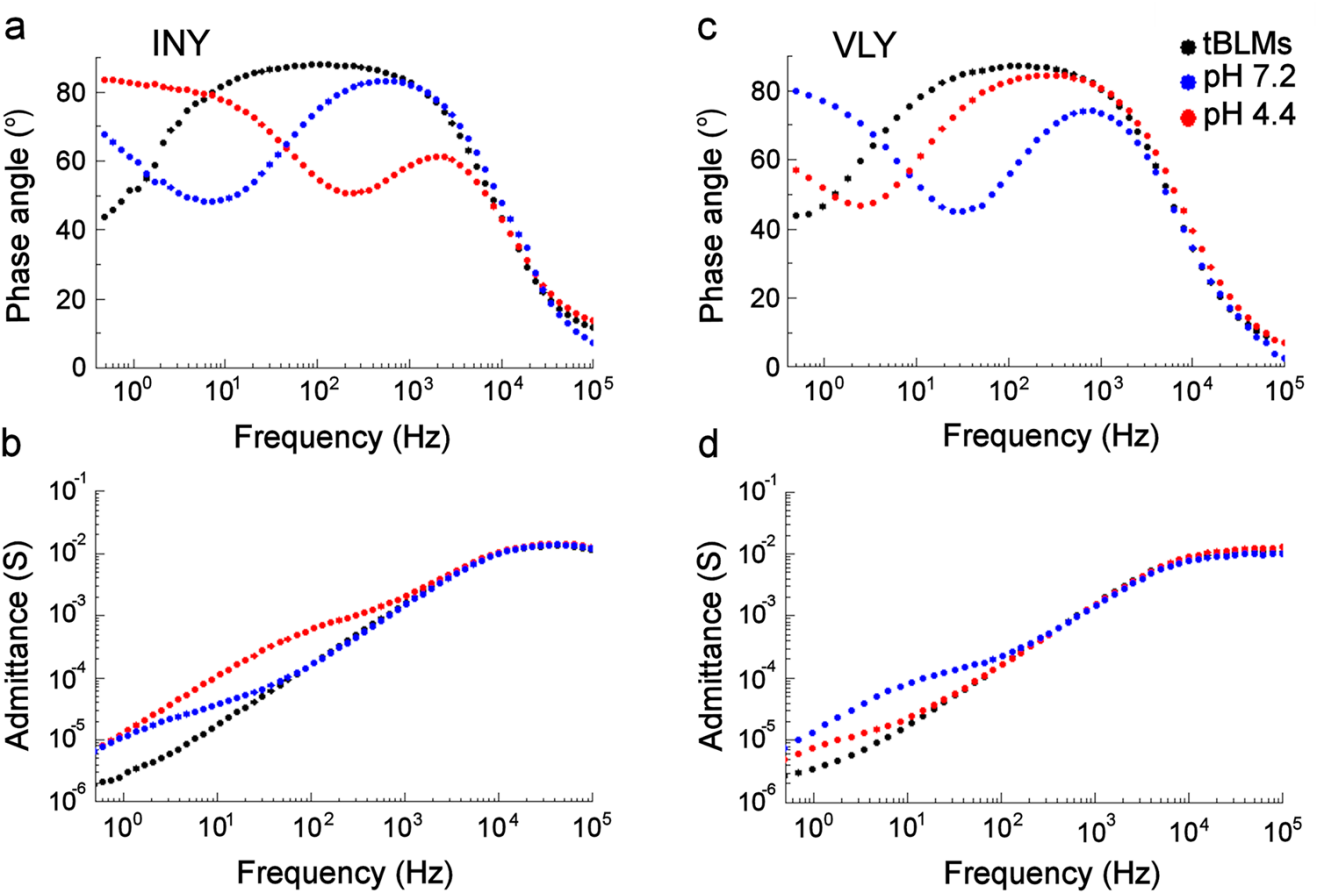
Bode plots representing INY (**a**,**b**) and VLY (**c**,**d**) induced damage to tBLMs at different pH. The tBLMs containing DOPC/cholesterol at a molar ratio of 50/50 were treated with 1 nM of INY or VLY solutions at pH 4.4 (red line) and 7.2 (blue line). Exposure time 30 min. Electrical parameters of cholesterol-rich tBLMs without addition of toxins were indicated by a black line. (**a**,**c**) Admittance phase. (**b**,**d**) Admittance magnitude. The data were obtained from at least four independent experiments.

Typical EIS Bode spectra (in admittance representation) of pristine tBLMs composed of DOPC/cholesterol at a molar ratio of 50/50 exhibits a minimum of phase angle curve in the low frequency range (<0.5 Hz) (Fig. 2a,c, black curve) that coincides with the inflection point on the log admittance vs. log frequency scale (Fig. 2b,d, black curve)^22^. This feature attests for the presence of residual defects in tBLMs^22^.

The addition of pore-forming toxins into the tBLM bathing solution triggers specific changes in the EIS curves. The formation of pores in tBLMs shifts both the phase minimum and a step-like feature towards higher frequencies. As seen from Fig. 2, incubation of tBLMs with 1 nM INY at pH 4.4 for 30 min resulted in shifts of both features reaching ≈200 Hz (Fig. 2a,b, red line). Incubation of tBLMs with 1 nM VLY resulted in significantly smaller changes in the EIS spectra under the same conditions. The effect reverses at pH 7.2. Now VLY exerts noticeably stronger effect on the EIS curves than INY.

The shifts of EIS phase and magnitude can be used to evaluate defect densities in tBLMs^32^. In this work we used the equation allowing to approximately evaluate the density of defects^32^:

1 $$\mathrm{lg}({N}_{def})=0.934\,\mathrm{lg}({f}_{min})-0.953$$

where, *f*_*min*_ – the frequency of the phase minimum in Hz and *N*_def_ is the defect density in µm^−2^.

Calculations using Eq. (1) and the data from Fig. 2 showed that both toxins exert dialectric damage on tBLMs in a pH dependent manner. At pH 4.4, incubation of tBLMs with INY for 30 min produces 17.7 µm^−2^ while the density of VLY defects amounts only to 0.26 µm^−2^. The membrane-damaging effect is different at pH 7.2. Now, INY produces only 0.74 µm^−2^ while VLY generates 3.0 µm^−2^ of defects under the same incubation time (30 min). Importantly, the EIS changes were irreversible; they cannot be reversed by rinsing the cell with protein-free buffer. In contrast to the SPR experiment, the changes of EIS triggered by toxins at both 4.4 and 7.2 pH were permanent and they cannot be reversed/decreased by rinsing the measurement cell with protein-free buffer.

### Inerolysin-induced damage of tBLMs is cholesterol concentration-dependent

**Figure 3:**
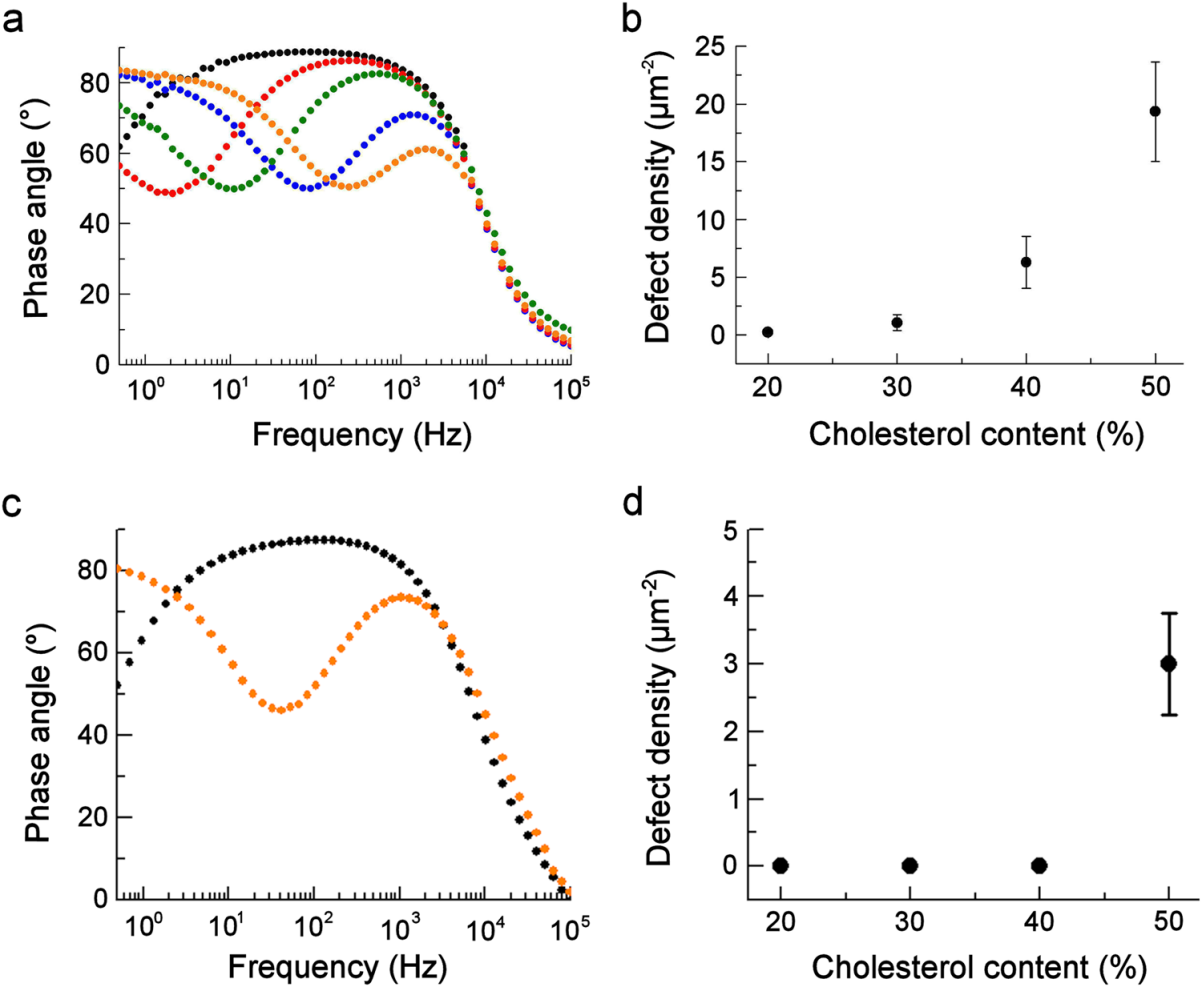
Bode plots representing INY (**a**) and VLY (**c**) induced damage to tBLMs composed of MLVs having different molar ratios of DOPC/Chol at pH 4.4 and 7.2, respectively. Exposure time 30 min. The EIS curves of different colors represent the respective DOPC/Chol ratio in MLVs: red = DOPC 80% + Chol 20%; green = DOPC 70% + Chol 30%; blue = DOPC 60% + Chol 40%; orange = DOPC 50% + Chol 50%; black = tBLMs without toxin. VLY did not induce damage in tBLMs containing 20–40% cholesterol; therefore, the data were not displayed. (**a**,**c**) Admittance phase. Dependence of INY (**b**) and VLY (**d**) induced defect density on cholesterol concentration in tBLMs. The data were obtained from at least four independent experiments.

**Figure 4:**
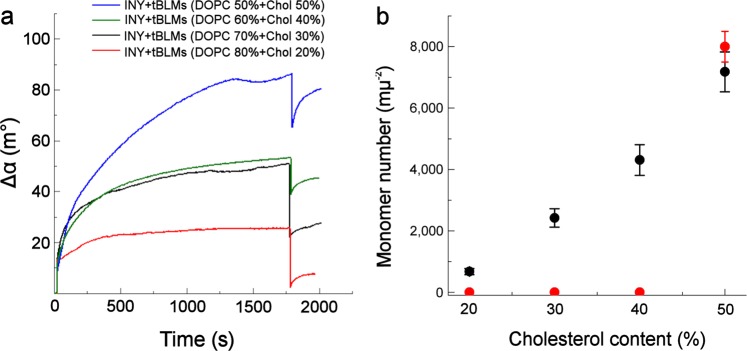
(**a**) The SPR angle shift produced by binding of INY (5 nM) to tBLMs containing various cholesterol concentrations at pH 4.4. (**b**) Dependence of monomeric INY (black dots) and VLY (red dots) binding on cholesterol content in tBLMs at pH 4.4 and 7.2, respectively. The SPR data obtained after rinsing the cell with protein-free buffer were used for calculations. The data were obtained from at least four independent experiments.

To determine the effect of cholesterol on the activity of INY, the tBLMs were prepared using different DOPC/cholesterol molar ratios: 80/20, 70/30, 60/40 and 50/50. The tBLMs containing various amounts of cholesterol were incubated for 30 min with 1 nM INY at pH 4.4. The resulting EIS curves are shown in Fig. 3. According to Eq. (1), the EIS admittance phase data were converted into the defect density. Both the EIS response and the defect density are continuously correlating with the fraction of cholesterol in tBLMs in the case of INY (Fig. 3a,b). However, for VLY we did not observe significant changes neither in the EIS (Fig. 3c,d) nor SPR (Fig. 4b) spectra for all membrane compositions with less than 40% of cholesterol. This observation is somehow at odds with earlier report on the reconstitution of VLY into tBLMs^30^. In this paper^30^ the authors observed a continuous increase of the EIS response for the composition below 40% and the stabilization of the membrane damage at 40% cholesterol and over.

It must be noticed that the straightforward comparison of the results reported in ref.^30^ is complicated as different techniques for the preparation of tBLMs were used in both studies. Our current technology involves a solvent-free fusion of multilamellar vesicles (MLVs) to a surface tailored with the molecular anchors. The authors in ref.^30^ used the ethanol based solvent exchange technique, which may involve partitioning of ethanol into the bilayer. Also, the exchange procedure may yield different lipid ratios in tBLMs, which may affect the observable activity of VLY. Different activities of VLY were indeed detected in tBLMs prepared using the solvent exchange and vesicle fusion methods. For example, the effect of 1 nM VLY on the EIS spectra, detected in this study (the tBLMs prepared using the MLVs fusion method) is by an order of a magnitude higher compared to the data in ref.^30^ (the solvent exchange method). The effect of the membrane composition on the activity of CDCs in general and VLY and INY in particular, is worth detailed investigation, but in this study, we focused only on the comparison of reconstitution of two proteins under the same conditions, including membrane composition.

INY demonstrated the cholesterol concentration-dependent binding to tBLMs monitored by SPR (Fig. 4). INY bound poorly to the lipid membrane containing low cholesterol concentration (Δα ≤ 10 m°), whereas the highest affinity was detected to tBLMs prepared from MLVs with 50 mol% cholesterol (Δα ≈ 80 m°).

According to the device configuration, the SPR sensitivity is 1 m° per 0.82 ng/cm^2^. This amount of protein corresponds to 90 monomers of INY per µm^2^. The binding of monomeric INY to tBLMs exhibits almost linear dependence on cholesterol concentration in tBLMs (Fig. 4b, black dots). However, binding of VLY to tBLMs was recorded only when 50% cholesterol concentration was reached (Fig. 4b, red dots).

### Visualization of cytolysins bound to cholesterol-rich tBLMs

**Figure 5:**
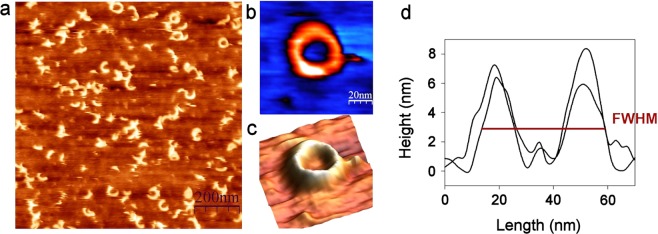
AFM topography images of tBLMs after incubation with 10 nM INY at pH 4.4. The tBLMs composed of MLVs containing DOPC/cholesterol at a molar ratio of 50/50. **(a)** Scan size is 1 μm × 1 μm. The enlarged 2D and 3D views of the ring-shaped defect, **(b)** and **(c)** respectively, scan size is 100 nm × 100 nm. (**d**) The profiles of perpendicular cross-sections from panel b. The width is measured as FWHM.

Visualization of oligomeric structures produced by toxins in cholesterol-rich tBLMs was performed using AFM. The SPR analysis showed that the shift of the SPR angle induced by VLY was obtained at a 4-fold higher concentration than that of INY (Fig. 1), therefore this concentration ratio of cytolysins was maintained in the AFM study. The tBLMs with cholesterol content of 50 mol% were incubated with 10 nM INY at pH 4.4 for 30 min, unbound protein was removed and typical membrane defects detected as ring-, arc- and slit-shaped structures were observed (Fig. 5a). The profile analysis of two perpendicular cross-sections (Fig. 5d) showed that the protrusion of the ring shaped defects was 7.1 ± 0.7 nm, that is consistent with the typical height of the inserted CDCs pore^33,34^. The inner and outer diameters of the complete pores of INY measured as the full width at half maximum (FWHM) were 23.2 ± 2.7 nm and 46.5 ± 4.1 nm, respectively (Fig. 5d). The obtained values are in line with the pore sizes of other CDC toxins suilysin and pyolysin^24,31^. The average height of the arc- or slit- shaped structures was 8.0 ± 1.0 nm suggesting that the majority of the observed incomplete assemblies are either inserted or partly inserted into the lipid bilayer.

**Figure 6:**
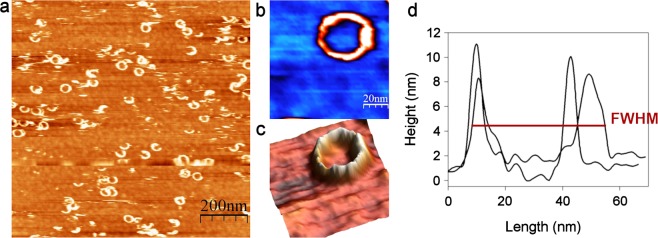
AFM topography images of tBLMs after incubation with 40 nM VLY at pH 7.2. The tBLMs composed of MLVs containing DOPC/cholesterol at a molar ratio of 50/50. **(a)** Scan size is 1 μm × 1 μm. The enlarged 2D and 3D views of the ring-shaped defect, **(b)** and **(c)** respectively scan size is 100 nm × 100 nm. (**d**) The profiles of perpendicular cross-sections from panel b. The width is measured as FWHM.

Incubation of 40 nM VLY with the cholesterol-rich tBLMs at pH 7.2 resulted in the ring-shaped structures that constitute the majority of the observed defects (Fig. 6). The average height of the ring-shaped defect was 7.5 ± 0.9 nm that is in line with the CDCs pore state. The inner and outer diameters of the complete pore of VLY measured as FWHM were 26.1 ± 2.7 nm and 50.3 ± 6.7 nm, respectively (Fig. 6d). The average height of all observed defects calculated from multiple images was 8.9 ± 1.8 nm. Incubation of VLY with cholesterol-rich tBLMs at pH 4.4 did not produce defects in the artificial membrane (Supplementary File S2) that is in agreement with the SPR results.

**Figure 7:**
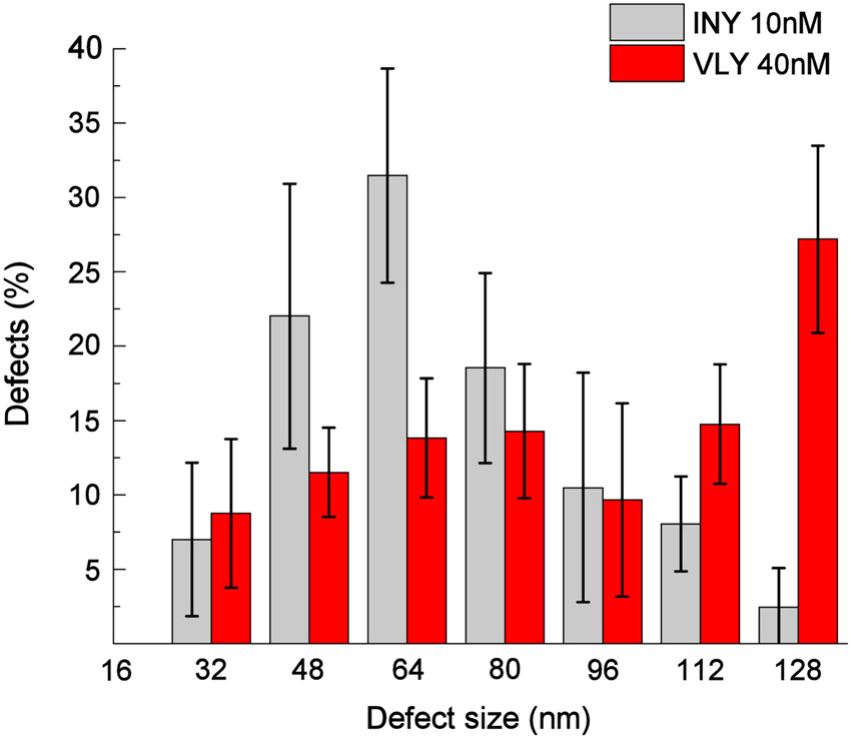
The size distribution of oligomeric structures obtained upon reconstitution of INY (grey bars) at pH 4.4 and VLY (red bars) at pH 7.2 into cholesterol-rich tBLMs detected by AFM. Nine AFM images (1 μm × 1 μm) were taken for analysis, each of them analyzed separately to count the defects. The size of defects is defined as the length of the rim of the oligomeric species (rings and arc-shaped structures, see Fig. 6 above) protruding above the tBLM surface.

Distribution of the observed assemblies expressed as the length (nm) of complete ring and incomplete arc-shaped structures obtained upon reconstitution of toxins into tBLMs is shown in Fig. 7. VLY mostly formed the complete rings, the length of those exceeded 112 nm. The broad peak of INY-generated defects centered in the range of 48–80 nm, and thus about 70% of the structures were detected as the arc-shaped entities. Only 2% of the defects corresponded to the complete ring structures (Fig. 7, grey bars), whereas this type constitutes >25% of the defects produced by VLY (Fig. 7, red bars).

## Discussion

The strict dependence on the presence of cholesterol in the target membranes needed to perform the pore-forming activity is the characteristic feature of CDCs^1^. Previous studies showed that VLY needs CD59 to accomplish its activity^13^, whereas INY is the classical CD59-independent CDC^16^. However, it was demonstrated that VLY is able to bind to and lyse cells lacking CD59^17^. The molecular basis for this dualism of VLY lays in the conformation of the loop containing the undecapeptide (UDP) in the tip of D4 domain^35^. The UDP-loop along with the L2 and L3 loops in D4 constitute the membrane binding interface and insert into the lipid bilayer anchoring the monomer to the surface^36^. The tip of this domain also contains the L1 loop, which situates the site for cholesterol recognition^4^. Analysis of the crystal structures of VLY bound to its receptor CD59 revealed that the UDP-loop can adopt at least two conformations. It can resemble either the conformation of strictly CD59-dependent ILY or adopt the structure of CD59-independend perfringolysin (PFO)^35^. The PFO-like UDP conformation allows conserved UDP arginine to form hydrogen bonds with the L1 loop, that precondition efficient L1 recognition and binding to cholesterol embedded in the membrane^35^. The ILY-like conformation that adopts the UDP-loop does not require orienting in a proper conformation the L1 loop, as ILY initially binds to membrane via CD59. The ability of VLY to adopt the PFO-like UDP conformation allows bypassing the assistance of CD59 to execute its pore-forming activity^35^. Thus, this VLY feature allows using tBLMs as a model for characterization of VLY interaction with membranes. Although this model does not closely resemblein *vivo* conditions, the studies herein have demonstrated biological relevance of the interaction between CDCs and tBLMs.

In this work, we examined reconstitution of INY and VLY produced by vaginal bacteria into artificial lipid membranes at different pH and cholesterol levels using EIS, SPR and AFM. The ability of INY from *L. iners* to impair the integrity of the phospholipid membranes was detected by the EI spectral changes. The increase in the admittance magnitude and the shift of admittance phase minimum in the frequency range from <0.5 to 100 Hz demonstrated the loss of insulating properties of tBLMs and populating membrane with ion-conducting defects in the phospholipid membranes^32^. The obtained EIS changes are irreversible and cannot be restored by removing INY from the membrane bathing medium. This type of EI spectral characteristics is common for other pore-forming toxins^37,38^.

Changes in the EIS signatures linking to INY and VLY-induced damage of tBLMs were confirmed by the AFM lipid surface images showing the oligomeric assemblies of toxins (complete and incomplete rings). It has been established that the CDC β-barrel pore has a characteristic protrusion by typically 7–8 nm of the oligomeric entities above the membrane^24,33^. The height of about 11 nm above the membrane surface indicates the membrane-associated prepore state without protrusion into bilayer^24,33^. Calculations of the height of oligomeric structures from the AFM data demonstrated that the complete rings and the majority of incomplete arcs generated by INY and VLY perforate the bilayer and form a water-filled pore. The size of defects showed that VLY formed a siginificant amount of complete rings, whereas in case of INY prevailed the incomplete arc-shaped structures. The arcs and slits perforating membrane form smaller size pores that that of complete rings^24,39^. However, the role of these arciform pores in the mechanism of pathogenesis is not without controversy. Another CDC member, listeriolysin (LLO), generated lessons of different pore size under native conditions that is probably due to involvment of this toxin in a variety of the pathways in the cell^40,41^. The data on kinetics of CDCs activity measured for pneumolysin from *S. pneumonia* suggested that formation of incomplete pores are affected by protein concentration and other reaction conditions^42^. Thus, it has to be determined whether membrane perforation by the arc-shaped complexes of VLY and INY is formed under native conditions.

It was known the capability of CDCs to bind to and induce damage of the membranes with a high content of cholesterol (>30 mol%)^3,43,44^. The data of INY binding to tBLMs and the estimated INY-induced defect density in the bilayer revealed the direct dependence on cholesterol concentration in contrast with that of observed for VLY. Both the EIS and the SPR data revealed, that VLY binding to tBLMs and its impact on the membrane integrity demonstrated no direct dependence on the cholesterol concentration in the bilayer: the effect was detected on tBLMs with high cholesterol content (50 mol%). Moreover, binding of VLY to tBLMs containing 50 mol% cholesterol was confirmed by other methods: the SDS-agarose electrophoresis demonstrated a sharp transition from undetectable binding of VLY to liposomes with less than 40 mol% cholesterol to maximal binding at 55 mol% total membrane cholesterol^17^. On contrary, INY showed its activity on the artificial membranes with low cholesterol content (20–30%). It was demonstrated that CDCs display differences in binding to cholesterol-rich membranes^2,45^. The lipid environment that modulates packing of the membrane may also have an impact on CDCs binding specificity to cholesterol^45^. It has been recently reported that CDCs binding properties could be realized by altering the structure of the L3 loop in D4. A single point mutation in this loop increased binding of PFO to artificial and natural membranes^45^. We hypothesize that the effect of INY to the tBLMs with low cholesterol content is connected with the structure of the L2 and L3 loops. Experimental evidence is needed to support this idea. The studies demonstrated that the mechanisms evolved by CDCs to target membranes with different cholesterol content allows microorganims to perform specific functions at healthy and disease state^2,45,46^. Recently, a 3-dimensional model of vaginal epithelium that resembles *in vivo* model has been employed to analyze the VLY activity in relation to tissue polarity^18^. Surprisingly, VLY did not affect cells in the apical side, but it permeabilized cells then exposed basolaterally. The detected lower concentration of CD59 in the apical than in the basolateral side of this tissue model may explain variation in the activity of VLY^18^. However, the differences in membrane cholesterol content embedded in membranes could be also an issue not analyzed by Garcia and colleagues^18^.

The biological relevance of the interaction of toxins and tBLMs was verified analyzing the activity of INY and VLY at different pH. The hemolytic activity of these toxins on mammalian erythrocytes is pH-dependent^16^. INY expressed its maximal activity at acidic pH and lost activity at neutral pH. On contrary, VLY exhibited a neutral pH optimum with weak or no activity at acidic pH. We did not observe unfolding of VLY and INY proteins in the pH range where toxins expressed their marginal activity, whereas the loss of LLO activity at pH 7.4 is related to protein denaturation^16,47^. The effect on the tBLMs insulating properties correlated with the pH optimum of the toxins: the INY-induced defect density on tBLMs was clearly higher at acidic pH, whereas VLY exhibited a significant effect on the integrity of tBLMs at pH 7.2. The AFM data revealed that no VLY-induced membrane defects were detected at acidic pH.

Inerolysin binding to tBLMs detected by SPR was also pH-dependent that contradicts recent findings by Rampersaud *et al*.^48^. The authors showed that the initial INY membrane binding and oligomerization is pH-independent, whereas membrane insertion step is defective at neutral pH^48^. However, we found that INY exhibited weak or no binding to tBLMs at neutral pH. We also demonstrated that VLY binding to the bilayer was pH-sensitive: the toxin binds to tBLMs and displays activity optimum on the cholesterol-rich artificial membranes and natural cells at neutral pH. The obtained results might have clinical importance. The normal pH in the vagina of reproductive-aged woman is in the range 3.8–4.4^28^, where VLY, if *G. vaginalis* secreted, is inactive. *G. vaginalis* has been recovered from the vaginal samples of almost all women with bacterial vaginosis^27,49,50^, a form of dysbiosis, characterized by an elevated vaginal pH^28^, which is optimal for VLY activity. *L. iners* has been found one of the most frequently isolated organisms in the vagina^25,26^. The presence of *L. iners* in healthy and dysbiosis conditions demonstrates its adaptation that was confirmed by the analysis of the complete genome sequence^51^. It was found that *L. iners* is a dominant species in the transitional state between healthy and abnormal vaginal microbiota and during menses^26^. The profile of INY activity might have an impact to *L. iners* adaptation to these particular vaginal conditions. Our study demonstrated that the tBLMs methodology allowed detecting toxins expressing their activity at different pH. The obtained results broaden the possibilities of tBLMs in bioanalytical applications. Bacteria *G. vaginalis* and *L. iners* inhabit the same ecological niche and present simultaneously at certain vaginal conditions, therefore the tBLMs approach would elucidate the presence and activity of CDCs in biological samples.

## Methods

### Generation of inerolysin

DNA fragment containing the INY coding gene lacking the signal sequence was amplified using Phusion proofreading polymerase (Thermo Fisher Scientific, Vilnius, Lithuania) from genomic DNA extracted from a vaginal swab of woman found *L. iners* positive^27^. A vaginal swab was obtained from the study approved by the Lithuanian Bioethics Committee (Approval No. 158200-1-3697-223, 12/11/2013, Amendment No. 2, 8/12/2015). Written informed consent was obtained from all study participants prior to enrollment. Amplification was performed using primers described in the previous report^16^. The PCR fragment was subjected to sequencing and cloned into pET28a(+) vector (Novagen/Merck kGaA). The resulting plasmid was transformed into *E. coli Tuner (DE3*) strain (Novagen/Merck kGaA). Recombinant N-terminally-hexahistidine tagged INY was purified using affinity chromatography by Ni-Sepharose 6Fast Flow (GE Helthcare Bio-Sciences, Uppsala, Sweden) according to manufacturer’s recommendations. The purified recombinant protein was stored in PBS (pH 7.4) supplemented with 1 mM DTT.

### Determination of hemolytic activity of inerolysin and vaginolysin

The use of human erythrocytes from healthy adult volunteer followed written informed consent was approved by the Council of Life Science Center of Vilnius University (Protocol of 21/11/2018, no. 600000-TP-10) and was performed in accordance with the standards of the Helsinki declaration. The hemolytic activity of INY and VLY was determined on human erythrocytes as described previously^52^ with some modifications. Erythrocytes were washed and stored in sterile PBS (pH 7.2). Toxins were diluted in the buffer containing 125 mM NaCl besides 35 mM pH buffer (sodium phosphate at pH 6.0, 6.5, 7.0, and 7.5 and sodium acetate at pH 4.5, 5.0, and 5.5) and kept on ice at least for 30 min before the assay. INY dilution buffer contained 2 mM of DTT^16^. The hemolytic activity of each toxin was performed in the toxin’s dilution buffer having a respective pH. The HD_50_ was defined as the concentration of toxin required lysing 50% of human erythrocytes.

### A deposition of gold film

Gold films were deposited onto the following substrates: BK7 glass slides (25 mm diameter, 1 mm thickness, AutoLab, Methorm, The Netherlands) for surface plasmon resonance (SPR), glass slides (25 by 75 mm (Thermo Fischer Scientific, UK) for electrochemical impedance spectroscopy (EIS) and freshly cleaved mica sheets (V-4 grade, SPI Supplies Inc, USA) for atomic force microscopy (AFM). Before vacuum deposition of metals, the substrates, except mica, were cleaned in sulfuric acid, rinsed thoroughly with a copious amount of deionized (18.2 Ohm/cm^2^, Mili-Q, Millipore) water and dried in a dry stream of nitrogen (99.99%). The films of chromium (~1 nm) and gold (~50 nm for SPR and AFM, ~80 nm for EIS) were coated using the PVD 75 (Kurt J. Lesker Company, USA) magnetron sputtering system. Sputtering parameters for 2 inches’ diameter metal targets were, as follows: Cr, power 200 W at 4.5 mTorr argon pressure; Au, power 200 W, sputtering current at 3 mTorr argon pressure. Prior to coating, the deposition chamber was evacuated to <10^−7^ mTorr of the residual pressure. Ultrahigh purity and scientific grade argon (AGA, Sweden) was used for plasma sputtering.

### Preparation of multilamellar vesicles

The formation of tBLMs is a multistep process that includes the deposition of an anchoring self-assembled monolayer (SAM), preparation of MLVs and the fusion of these MLVs to the anchoring SAM. Vesicle suspensions were prepared from 1, 2–dioleoyl–sn–glycero–3–phosphocholine (DOPC) and cholesterol from Avanti Polar Lipids Inc. (USA) at various molar rates as described previously^53^. Briefly, the lipids were dissolved in 99% chloroform (Sigma-Aldrich) to a concentration of 10 mM. An aliquot of this lipid solution was transferred to a separate vial and chloroform was evaporated under a continuous flow of nitrogen until a thin, uniform lipid film formed (~30 min). The dried lipid film was resuspended in the buffer solution (0.1 M NaCl, 0.01 M sodium acetate buffer, pH 4.4) to a 1 mM total lipid concentration using an automatic pipette (1 mL tip) and slowly (1 cycle/s) aspirated and dispensed until the lipid film on the vial walls dispersed and the solution became milky (~100 aspiration-dispense cycles to obtain a homogenous, though, milky opaque preparation). Thus, the prepared MLVs can be stored at +4 ºC temperature for 1 month. However, before each experiment the lipid mixture in the buffer must be mixed again (usually 20–30 aspiration-dispense cycles) to fully restore their functional properties.

### Preparation of tethered bilayer membranes

The tBLMs were prepared as describe previously^53^. Briefly, freshly gold-coated substrates were immersed in 0.05 mM ethanolic solution of the tether WC14 (20–tetradecyloxy-3,6,9,12,15,18,22–heptaoxahexatricontane–1–thiol; synthesized in–house), and β-mercaptoethanol (ßME) (Sigma-Aldrich, St. Louis, MO) mixed at molar ratio 30:70 to form mixed self-assembled monolayers (SAMs)^20^. Incubation of the gold substrates in the WC14/βME solutions was carried out for 10–12 h. Then the substrates were washed in pure ethanol and dried in a stream of nitrogen. Samples with the self-assembled monolayers were exposed to a multilamellar vesicles solution (vide infra) for up to a 30 min during which tBLMs were formed. The formed bilayers exhibited stable electrical and optical parameters for at least 72 h. Before addition of toxin, the tBLMs were washed and the cell was filled with the buffer containing 0.1 M NaCl and either 10 mM sodium acetate (pH 4.4) or 10 mM sodium phosphate (pH 7.2). Where indicated, the respective buffer was used to dilute toxins; the buffer for INY contained 2 mM of DTT. The control experiment showed no effect of DTT on the integrity and electrochemical properties of tBLMs.

### Electrochemical impedance measurements

Electrochemical impedance (EI) was measured using an electrochemical workstation Zennium (Zahner, Kronach, Germany) in the frequency range between 0.5 Hz and 100 kHz with 10 logarithmically distributed measurement points per decade. A saturated silver–silver chloride (Ag/AgCl/NaCl (aq. sat.) microelectrode (M – 401F, Microelectrodes, Bedford, NH), used as a reference, has the potential + 196 mV vs. standard hydrogen electrode while the auxiliary electrode was a platinum wire (99.99% purity, Aldrich; d = 0.25 mm) coiled around the barrel of the reference electrode. Measurements were carried out using 10 mV alternating current at 0 V bias versus the reference electrode in aerated solutions. The EIS measurement cell holder contained 6 separate vials (working surface area is 0.32 cm^2^ of each vial) that allowed carrying out 6 individual experiments on the same solid substrate with the identical lipid compositions for both VLY and INY tests. The effect of toxins at the concentration of 1 nM on tBLMs was determined after 30 min of incubation at room temperature.

### Surface plasmon resonance

The SPR measurements were conducted on an Autolab Twingle system (Eco Chemie B.V., The Netherlands) equipped with a flow-through cell having a volume of 175 μL. The unit performs the SPR spectra recording at a fixed wavelength of 670 nm. It automatically follows with a millidegree resolution the position of an incidence angle (ranging from 62 to 78°), at which minimum of the reflection due to an excitation of SPR is observed. Half cilinder prism BK7 with a 1.518 refractive index was used in the SPR measurements. Model F34 refrigerating/heating circulator (Julabo, DEU) was used to stabilize temperature at 21 ± 0.1 °C. Before each experiment, the baseline in the respective buffer was recorded. Before addition of toxin, the cell was equilibrated with the buffer containing 0.1 M NaCl and either 10 mM sodium acetate (pH 4.4) or 10 mM sodium phosphate (pH 7.2). The protein-tBLMs interaction was started to record immediately after protein injection. After 30 min the cell is flushed with the respective protein-free buffer to remove unbounded protein. All measurements were carried out at stopped-flow conditions.

### Atomic force microscopy imaging and image processing

The post-action imaging of incorporated protein in the tBLMs was carried out by AFM in a tapping mode on a Dimension Icon (Bruker, Santa Barbara, CA, USA) scanning probe microscope system at a room temperature (20 ± 0.5 °C) using triangular SNL–C probes (Bruker, Santa Barbara, CA, USA). The tBLMs used for AFM were composed of MLVs containing DOPC/cholesterol at a molar ration of 50/50. The tBLMs were repeatedly rinsed with the sodium acetate (pH 4.4) or phosphate (pH 7.2) buffer. The buffer solution containing 10 nM INY or 40 nM VLY was added to the imaging cell and the tBLMs sample was subjected to the toxin-mediated activity for 30 min. Prior the imaging the cell was flushed with the protein-free buffer. During the whole process, the mica surface was not allowed to become dry. The multiple images of different scanning areas and angles were taken for each sample. Typically, the scans were performed at 512 pixels or higher resolution with a scan rate of 0.5–1 Hz. Images were analyzed by scanning probe microscopy software (WSxM)^54^. The length and profiles of defects were tracked and analyzed manually by open source software ImageJ (https://imagej.nih.gov/ij/). The AFM tip radius has not been excluded from measured value.

### Statistical analysis

The hemolytic activity data are presented as the arithmetic mean (SD) of at least three independent experiments performed in duplicate. The SPR and EIS data are expressed as the arithmetic mean (SD) of at least four independent experiments. Error bars in the plots represented standard deviation of the mean. Statistical analysis was performed using OriginPro 8.5.

## Supplementary information

Supplementary information

## Data Availability

All data generated or analyzed during this study are included in this article and its Supplementary Information Files.
