# Supplementary material for: Inerolysin and vaginolysin, the cytolysins implicated in vaginal dysbiosis, differently impair molecular integrity of phospholipid membranes
Source: Sci Rep. 2019 Jul 23;9:10606. doi: 10.1038/s41598-019-47043-5 (PMC6650466; doi:10.1038/s41598-019-47043-5)
Supplement: Supplementary file 1 — Supplementary information [file 41598_2019_47043_MOESM1_ESM.pdf]

Supplementary information

Inerolysin and vaginolysin, the cytolysins implicated in vaginal dysbiosis, differently impair molecular integrity of phospholipid membranes

Tadas Ragaliauskas, Milda Plečkaitytė, Marija Jankunec, Linas Labanauskas, Lina Baranauskiene, and Gintaras Valinčius

Supplementary File S1

Protein stability analysis using fluorescence-based thermal shift assay

Protein stability was analyzed using fluorescence-based thermal shift assay (FTSA, also known as differential scanning fluorimetry) [1-3]. The protein was heated at a constant rate and unfolding was studied using extrinsic fluorescence of the solvatochromic probe 8-anilino-1-naphtalensulphonate (ANS). Experiments were performed in Corbett Rotor-Gene 6000. Fluorescence intensity was monitored using the blue channel for excitation (365±20 nm) and detection (460±15 nm) with a heating rate of 1°C/min. The samples contained 0.2 mg/mL of purified INY or VLY, 100 mM of the respective buffer, and 100 µM ANS. To exclude buffer-specific effects protein stability was tested in the pH range 3-12 using the following buffers: HEPES (4-(2-Hydroxyethyl)piperazine-1-ethanesulfonic acid), MES (2-(N-Morpholino)ethanesulfonic acid), MOPS (3-(N-Morpholino)propanesulfonic acid), TRIS (2-Amino-2-(hydroxymethyl)propane-1,3-diol), acetate, citrate, phosphate, and universal buffer mix (composed of 50 mM acetate, 50 mM phosphate and 25 mM borate). Fluorescence intensity curves were recorded to determine the melting temperature T_m (the inflection point of the melting curve) where the protein is considered to be half folded and half unfolded [4]. Both INY and VLY remain stable molecules under the acidic conditions in the temperature range 30-38°C: the detected T_m at pH 4.3 for INY and VLY were 43°C and 44.3°C, respectively (C and D panels). At pH 6.0 and 7.0, unfolding was observed when both proteins were subjected to 48-55°C.

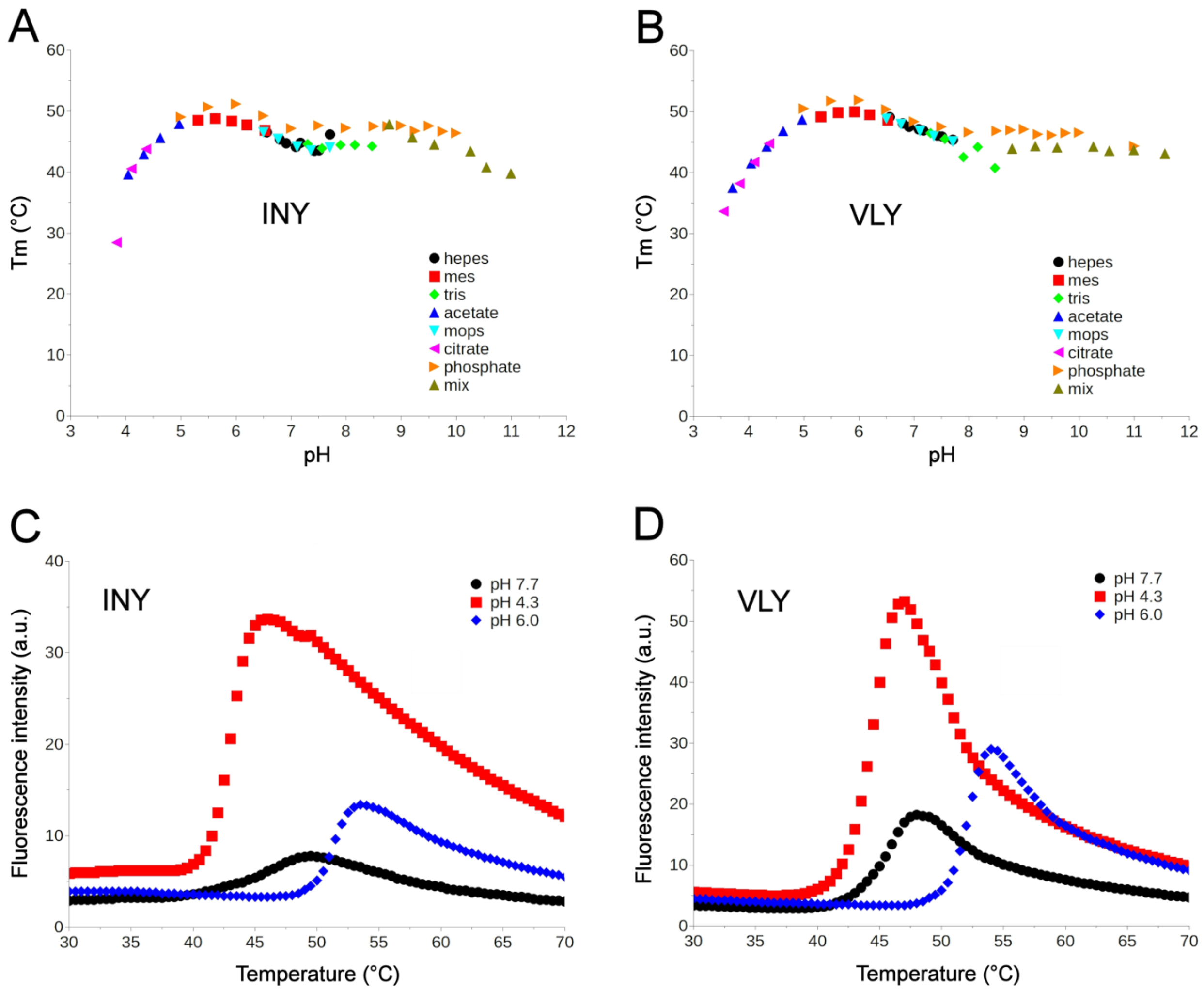

(A, B) Stability of INY and VLY in the pH range 3-12. (C, D) Unfolding curves of INY and VLY at different pH.

References

1. Pantoliano, M. W. *et al*. High-density miniaturized thermal shift assays as a general strategy for drug discovery. *J. Biomol. Screen*., **6**, 429–440 (2001).
2. Lo, M. C. *et al*. Evaluation of fluorescence-based thermal shift assays for hit identification in drug discovery. *Anal. Biochem*. **332**, 153– 159 (2004).
3. Simeonov, A. Recent developments in the use of differential scanning fluorometry in protein and small molecule discovery and characterization. *Expert. Opin. Drug Discov*. **8**, 1071–1082 (2013)**,** https://doi: 10.1517/17460441.2013.806479.
4. Matulis, D., Kranz, J. K., Salemme, F. R. & Todd, M. J. Thermodynamic stability of carbonic anhydrase: measurements of binding affinity and stoichiometry using ThermoFluor. *Biochemistry*, **44**, 5258–5266 (2005).

Supplementary Figure S1

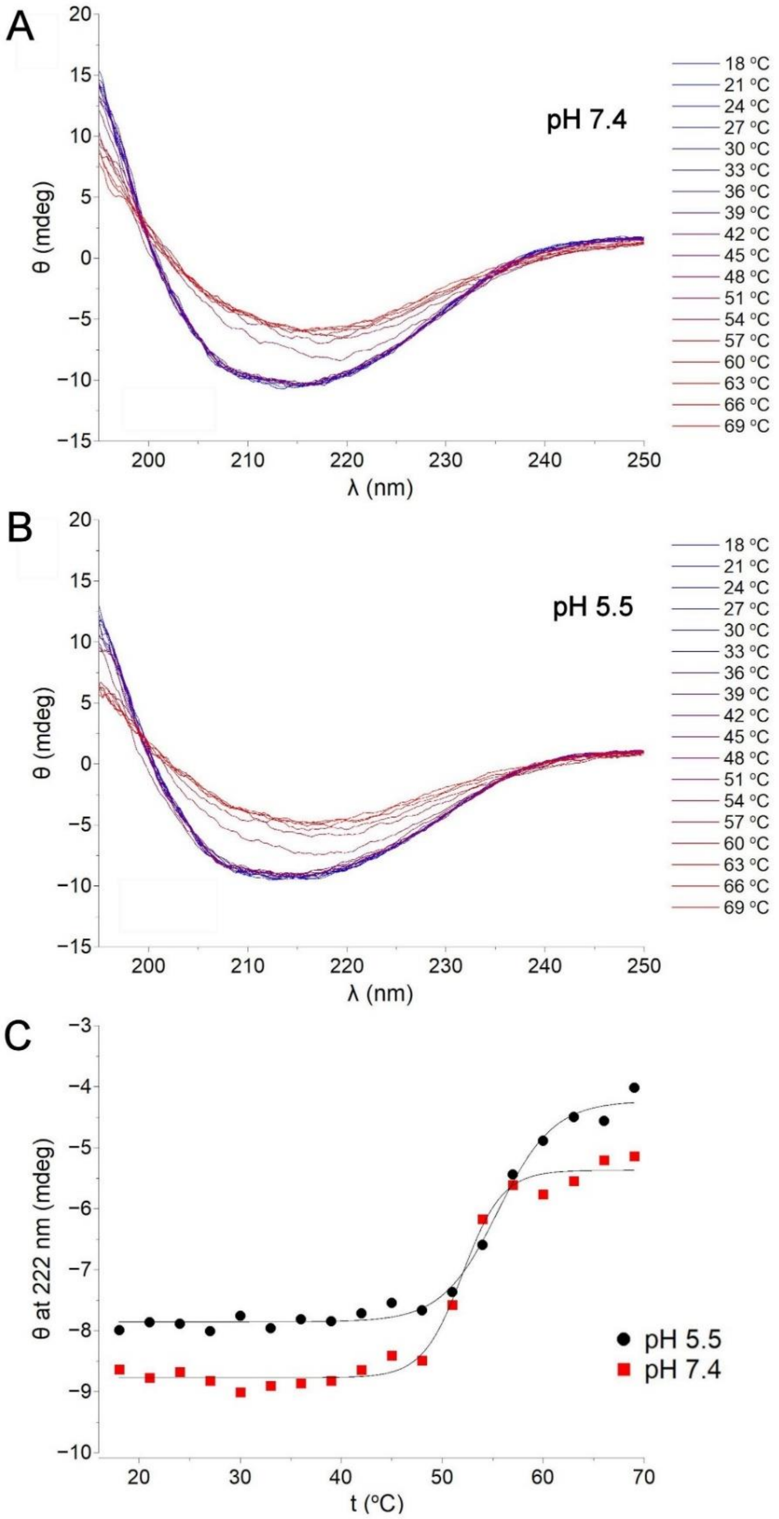

Figure S1. The raw CD spectra of INY recorded at different temperatures in the buffer solutions with pH 7.4 (A) and 5.5 (B). Protein concentration is 0.1 mg/mL. (C) The obtained *Tm* values (52.0°C at pH 7.4 and 55.5°C at pH 5.5) are in good agreement with that of obtained with the FTSA method.

Supplementary File S2

Visualization of the effect of VLY on cholesterol-rich tBLMs at pH 4.4

The cholesterol-rich tBLMs were incubated with 40 nM VLY at pH 4.4 for 30 min. The unbound protein was washed out with the protein-free buffer solution (pH 4.4.). AFM performed in protein-free buffer. As seen in the figure below no defects in tBLMs were detected.

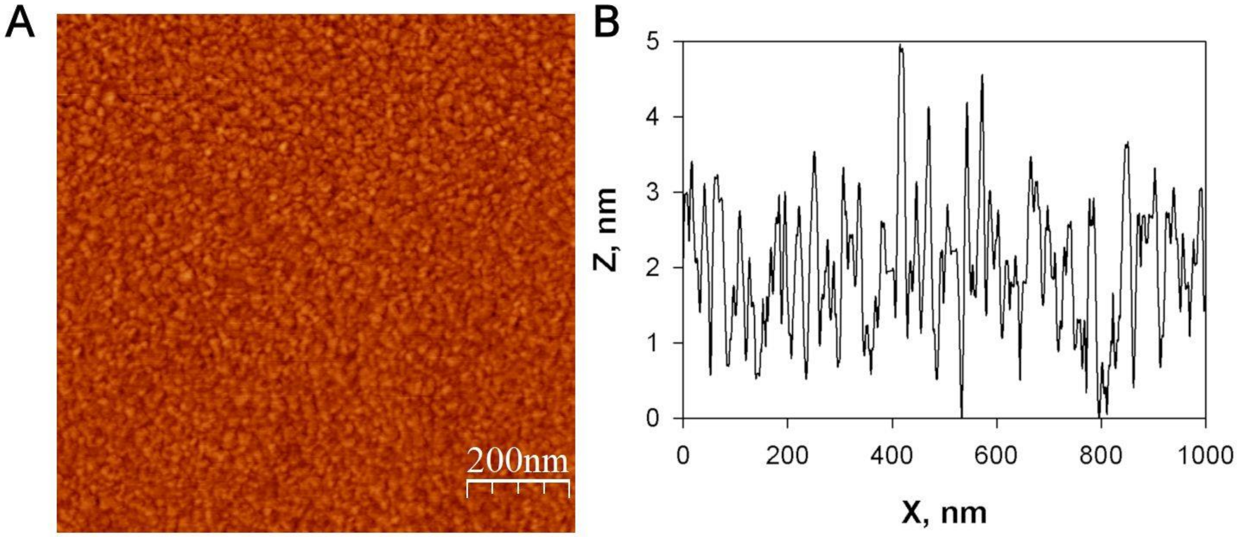

AFM topography image (A) and horizontal profile (B) of tBLMs after incubation with 40 nM VLY. tBLMs composed from MLVs containing DOPC/cholesterol at a molar ratio of 50/50 and pH 4.4. Scan size is 1 µm x 1 µm. Z and X indicate height and length, respectively.
